# Communities and community genetics in Ethiopia

**DOI:** 10.11604/pamj.2014.18.115.3172

**Published:** 2014-06-05

**Authors:** Luche Tadesse, Fikru Tafesse, Hanan Hamamy

**Affiliations:** 1 Tehran University of Medical Sciences, Iran; 2 Freelancer, Addis Ababa, Ethiopia; 3 International Rescue Committee, Jordan; 4 Geneva University, Switzerland

**Keywords:** Genetic, disorders, diseases, birth defects, congenital, community, Ethiopia

## Abstract

The rates of congenital and genetic disorders in low and middle income countries are similar or might be higher than in high income countries due to a multitude of risk factors and the dearth of community genetic services. To direct effective preventive, diagnostic and counseling services, collecting data on the incidence and prevalence of various congenital and genetic disorders and their risk factors is a pre-requisite for establishing genetic services at the community level and mainly at the primary health care setting. This brief review is meant to assess the available epidemiological data in Ethiopia pertaining to congenital and genetic disorders on which the future community genetic services could be built. Existing epidemiological data on congenital and genetic disorders in Ethiopia is limited, and the few studies conducted revealed that folate and iodine deficiencies are prevalent among women in the reproductive age. Pregnant women's infection with syphilis and rubella is prevailing. Based on available data, cleft lip and palate, congenital heart diseases, club-foot, and gastro-intestinalmalformations are the most common birth defects in Ethiopia. Community based studies to accurately demonstrate the incidence and prevalence levels of these disorders are almost unavailable. To plan for organization and implementation of community genetic services at the primary health care level in Ethiopia, conducting standardized epidemiological studies is currently highly recommended.

## Introduction

Ethiopia is located in Eastern part of Africa and is one of the highly populated countries in Africa with a geographical area of 1.14 Million Square Kilometers [1]. The population census in Ethiopia is being conducted every ten years, and the last census conducted was in 2007 where the total population of Ethiopia amounted to 73, 750, 932 (50.5% male and 49.5% female population) [2]. The official projection of population by July 2013 is 86,613,986,84% of the total population is residing in the rural region [3].

Since 1995, Ethiopia is a “Federal Democratic Republic” country, consisting of nine regional states and two chartered city administrations, namely Addis Ababa and Dire Dawa cities [4]. The largest regional state in the country is Oromia where 37.2% of population resides, followed by Amhara region and Southern Nations and Nationalities Region, accounting for 22.2% and 20.7% of the total residents, respectively. The rest of the populations occupy the other six remaining regional states and two city administrations. In terms of religion, 43.5% of Ethiopian population is Orthodox Christian, 33.9% Muslim, 18.6% Protestant, 2.6% Traditional, 0.7% Catholic and 0.7% other religions [5].

With an aim of accessing all people with health services, Ethiopian Federal Ministry of Health/EFMOH has changed the previously existing six-tier health care delivery system into four, and then more recently into three-tier health care delivery system. The current system has three layers: the first layer at the bottom is a Primary Health Care Unit/PHCU (formed by a primary hospital, a health center supervising as its catchments five health posts), which is intended to serve a maximum of 100, 000 population; the second layer from the bottom is General Hospital that serves up to 1.5 million people and it is a referral site for PHCU; and the third is Specialized Hospital, serving around 5 million people and it serves as referral center for the second layer and responsible for teaching of health professionals [6]. Tiers were reduced to de-centralize the health services and make them accessible to the rural community at large. PHCU includes a primary hospital serving from 60,000 to 100, 000 people,a Health Center/HC that provides services to 25, 000 people,and a Health Post/HP serving a maximum of 5,000 rural people. The Health post is staffed by two health extension workers, who have at least completed 10 years of education plus additional year of training in basic primary health care service with emphasis on preventive health care [7].

As health insurance is unavailable for workforce in the public sectors and rural community, out of pocket money share is 37%to purchase health care in Ethiopia. The government's contribution is 54%, and the rest 9% is covered by other sources [8]. However, the government plans to introduce community health insurance and social insurance, which is currently under way [6].

The current focus of Ethiopian Ministry of Health is on controlling communicable diseases at the primary health care units and there are no strategies or policies focusing on community genetic services. Thus, in Ethiopia, studies and services related to human genetics are yet to fully emerge and currently little information on genetic disorders, and community genetics and services exist. This brief review aims to assess the situation of congenital disorders/birth defects and available genetic services based on existing epidemiological studies in the country.

## Methods

Methods used for this purpose has been an extensive literature search for on-line publications through accessing PubMed, Google Scholar, World Health organization (WHO) publications as well as data recruited from research institutions and universities in Ethiopia. The key words used during online search include: “genetic”, “disorders”, “diseases”, “birth defects”, “syphilis”, “Ethiopia”, “congenital diseases/disorders,” “teratogen”, “pregnancy”, “sickle”, “hypothyroidism”, and sometimes by combining each of the above words as a phrase. As the number of available studies on genetics in Ethiopia is very limited, all the full articles obtained were taken into consideration for review without applying any exclusion or inclusion criteria.

## Current status of knowledge

### Current Population

Ethiopia is a multi-ethnic country with different cultural identities and languages. Based on the national census of 2007, the total national population consisted of 35% of Oromo, 27% of Amhara, 6.2% of Somali, 6.1% of Tigrean, 4% of Sidama, 2.5% of Gurage, 2.3% of Welayta, 1.7% of Hadiya, 1.7% of Afar, 1.5% of Gamo, and others [2]. In Ethiopia, there are currently over 80 languages spoken by various ethnic groups including 22 Cushitic, 12 Semitic, 18 Omotic, and 18 Nilo-Saharan [9]. There have been a number of genetic studies focusing on the origin of Ethiopian people with no strict and clear evidence existing about the origin of Ethiopian population. Ethiopian people are speculated to be a mixed race, ranging from African race to Middle-East, Asia and in few studies reported of also having European origin. Ethiopian population have been reported to have “Caucasoid gene mainly through males” and African gene due to migration of Bantu to Sub-Saharan Africa [10]. The Jewish origin is mainly the gene taken from the Ethiopian Jewish population and similarities have been reported between Ethiopian and Yemenites Jews [11]. Studies that analyzed the Y-chromosomal origin of Ethiopian population state the existence of Ethiopian gene out-of Africa, mainly from Asia [11, 12].

### Health Demographic Data

The median age at first marriage among women of reproductive age (15-49 years of age) is 16.5 years, whilst for the men the median age at first marriage is 23.1 years. Polygamy is common practice in Ethiopia, and 11% of women in marital-union report that their husbands have one or more other wives. The fertility rate in Ethiopia declined from 5.4 children per woman in 2005 to 4.8 children per woman in 2011, and is higher in rural areas (5.5 children per woman) than in urban areas (2.6 children per woman). In the capital city, Addis Ababa, the fertility rate is 1.5 children per woman. Factors that may influence the fertility rate include education, information about health services and residing in urban areas. The infant mortality rate is declining with a current rate of 59 per 1000 live births. The contraceptive prevalence rate among married couples is 29% and the unmet need for family planning services within married women is 25% [13, 14]. The life expectancy at birth in Ethiopia is 59 years for men and 62 years for women [15].

### Maternal Health Services and Status of Women in Society

In Ethiopia, the utilization of the key maternal health services like antenatal, natal and postnatal care services at the health facilities is generally low. Community based surveys demonstrated that between 2000 and 2005, only 27.4-28.5% of expectant mothers used the antenatal health care facilities [13, 16]. The marked improvement of antenatal care service users observed in 2011 (34%) could be explained by the introduction of Health Extension Program and expansion of health facilities, mainly primary health care units along with human resources development over the past five years in Ethiopia. Given that the rural population constitutes the main section of the population in Ethiopia, 90% of deliveries are actually home deliveries lacking the assistance of skilled health providers. Minimal increase in the number of deliveries attended by skilled health care workerswas seen over the last ten years; 5% in 2000, 6% in 2005, and 10% in 2011 [13, 14, 16]. Similarly, the postnatal care services utilization is very low with only 6% of mothers getting postnatal care from skilled providersand only 1% of women get services from Health Extension Workers [14].

Women in Ethiopia used to be at a disadvantage in the society with gender inequality in the accessibility to basic social services including education and health services, prevalence of domestic violence against women, presence of cultural and traditional factors that undermine women's right, widespread polygamy, and the heavy work-load imposed on women [17]. Education is currently considered by the government as a priority social service with focus on universal access to education. In 2010, the net enrolment of girls at primary school was 86.5%, whereas for boys it was 88% [18]. According to United Nations Educational, Scientific and Cultural Organization, the primary school enrollment status for the year ending 2009 was 85% for boys, and 80% for girls; and the secondary school enrolment for boys was 39% and for girls it was 30%, while the adult literacy rate for the year 2009 was 42% for men and only 18% for women [19]. Hence, though the primary school enrolment rate of girls is increasing noticeably, the dropout rate among female students is quite high and at secondary school the sex disparity between boys and girls is wide, and accessing the majority of Ethiopian women with education is yet to be realized. Despite the fact that the decision making power of women among the society is increasing with time due to access to education and economic empowerment of women, still the larger proportion of households decision making is in the hands of men [14].

Globally the practice of consanguineous marriages is related to culture and religion, and is most common among the Muslim community. Though in Ethiopia consanguineous marriages are believed to be common within the Muslim society which constitutes nearly 34% of the total Ethiopian population, there are no available published reports concerning the rates of consanguineous marriages in Ethiopia.

### Factors Predisposing to Congenital Disorders

Though most of the prevailing birth defects have a genetic origin, the interaction of genetic component with environmental factors in utero could also lead to congenital disorders, while some birth defects are chiefly secondary to environmental factors acting in utero. Early identification and treatment of infections before and during pregnancy can avoid the congenital disorders resulting from such infections as syphilis and rubella. Neural tube defects can be considerably prevented by pre-conceptional and dietary folic acid supplementation. Early newborn screening with proper management prevents the complications associated with iodine deficiency and congenital hypothyroidism [20]. Moreover, full history taking for family history of genetic diseases and counseling of parents could minimize the risks of birth disorders [21]. These measures could be implemented through the provision of genetic services at primary health care units. The problem is that the antenatal coverage by a skilled provider is only 34% [14] and the basic primary health care services are unavailable to all pregnant women.

Among congenital anomalies that could be prevented through dietary folate supplementation during pregnancy for women in reproductive age are the neural tube defects [22, 23]. These measures are under practiced in Ethiopia. A cross-sectional study conducted nationwide in Ethiopia shows that among women who participated in the study, 46% of them had severe folate deficiency [24]. Another potential risk factor for congenital disorder is Iodine Deficiency Disorder/IDD, which could be averted by iodization of salt in daily diet or supplementing Iodine tablets during pregnancy. In an extensive study conducted in Ethiopia nationwide among women aged 15-49, it was seen that the prevalence of goiter was35.8%, (95% CI, 34.5-37.1) with palpable gland in 24.3% and a visible goiter in 11.5% of women [17, 25].

On the other hand, despite inexistence of sufficient information on prescription of drugs,which have been proven to be associated with teratogenicity during pregnancy (specifically in the first trimester); a study conducted in the northern part of Ethiopia shows few drugs being prescribed “inappropriately” during pregnancy. These drugs were tetracycline and doxycycline in which both are believed to cross the placenta and cause permanent tooth discoloration, enamel hyperplasia and affect fetal skeletal growth. Other reported drugs inappropriately prescribed during pregnancy were cimetidine (causes gynecomastia in male fetus), and diclofenac (leads to neonatal pulmonary hypertension and hypertrophic cardiomyopathy) [26]. Published studies have indicated that substance abuse including alcohol consumption and smoking are associated with low birth weight, pre-term birth, fetal death, complications during pregnancy and labour and congenital anomalies to infants [27–29]. Use of alcohol and substances abuse such as khat chewing during pregnancy is on the rise in Ethiopia [30]. There are no available studies in the country that could elucidate the association of alcohol consumption and substance use with pregnancy outcomes.

Among sexually transmitted infections (STIs), infection with syphilis during pregnancy has been associated with congenital syphilis [31], spontaneous abortions, low birth weights and stillbirths [32]. Studies conducted in Ethiopia have shown the high level of syphilis infection among pregnant women; a hospital based study conducted in rural hospitals of Ethiopia reportedthat 13.7% of all pregnant women screened for syphilis had positive VDRL test [33]. According to the national Antenatal Care Sentinel Surveillance conducted in 2009 which was based on data taken from the antenatal care unitsthe prevalence of syphilis was 2.4%, with higher rates in rural (2.6%) than in urban (1.7%) facilities. The prevalence of syphilis among women in this study showed increasing levels with age ( 2% in the age group 15-24 years, 2.2% age for the group 25-34 years, and 2.8% for ages 35-49 years) [34]. However, these two studies may not reflect the true picture of syphilis in the general population due to the fact that a small proportion of pregnant women visit the health facilities for antenatal care, as only 34% of pregnant women in Ethiopia get ANC from a skilled health worker before delivery [14]. Moreover, those who are antenatal care service users are socio-demographically different from non-users and ANC service users tend to be urban residents and more likely to be educated. Therefore, the prevalence of syphilis could be higher than what has been reported and pregnant women in Ethiopia are at risk of giving birth to neonates with congenital syphilis and other complications.

Other studies conducted related to congenital disorders include the study on the rates of Rubella infection. The study revealed that among females18-25 years of age in the population residing in cities, the prevalence rates of rubella IgG antibody were 97% in Addis Ababa, 94% both in Northern (Dessie) and Southern part of Ethiopia (Awassa), 88% in Eastern part (Dire Dawa), and 85% in Western part (Gambella) [35]. There are two major areas of concern that could affect the validity of this study to generalize it to the national population. The first one is the age category used for this study was the relatively young and narrow of18 to 25 years. The study did not find any difference in rubella IgG antibody prevalence among the age groups. The second area of weakness is that the samples of population taken were from cities and the rural population was not included. The relatively remotely located city, Gambella (West of Ethiopia) showed a lower prevalence of rubella IgG antibody (88%) which might indicate the lower level of the rubella IgG antibody in the rural areas. The study might be overestimating the prevalence of the rubella IgG antibody in Ethiopia in general. However, this study [35] is informative for the risk factor of rubella in Ethiopian population, since to the best of our knowledge, no study has been conducted since then and the current level of the prevalence of rubella immunoglobulin (Ig) G antibody is unknown in the country. On the other hand, a longitudinal study conducted on all age groups of the population revealed higher prevalence of rubella cases (12.1%), among under-15 years of age (nearly 95%) of Ethiopian population. This study was based on data established for measles-case definition, and laboratory tests for rubella immunoglobulin (Ig)M was performed from these samples as there is no surveillance system for rubella in the country. Since measles is quite common among children; the case definition of measles mostly captures younger age population and higher prevalence of rubella among young people could be due to this. Moreover, rubella cases tend to be without any clinical signs and symptoms unlike measles [36]. Therefore, though this study could give the nationally representative information in terms of geographical coverage, under reporting of rubella cases might be possible due to the fact that sampling of specimen was based on the case-definition of measles.

A study carried out in Ethiopia among pregnant women reported that infection with malaria during pregnancy was associated with low birth weight and still-births, and the incidence of malaria among these pregnant women visiting antenatal clinics was 10.4% [37]. Hence, this is an alarming report as the malaria is endemic in most areas of the country and occurrence of epidemic of malaria is common every year after rainy season though various measures are being taken by Ministry of Health to prevent infection, treat cases promptly and control the epidemic when it happens.

Congenital Toxoplasmosis is another form of congenital disorder that is being transmitted to fetus from an infected mother throughout the period of pregnancy. Toxoplasmosis is asymptomatic in most cases and its transmission rate from mother to fetus increases with pregnancy. Some of the risk factors for infection of Toxoplasma Gondii are eating raw or under cooked meat, eating unwashed vegetables, contact with cat and soil [38]. Infection with Toxoplasmosis is also associated with the HIV status of individuals, as both HIV infection and pregnancy are believed to affect the immune system and such correlations have been reported in Ethiopia [39]. Available published and recent studies revealed that infection with Toxoplasma Gondii is common among child bearing age of women and pregnant women in Ethiopia. A study conducted in central part of Ethiopia among child bearing women showed that the sero-prevalence of toxoplasmosis of 81.4% (95% CI = 77.70, 85.13)-78.4% positive for IgG and 3.06% positive for both IgG and IgM antibodies. Furthermore, this study also reported the sero-prevalence of toxoplasmosis among pregnant women (213 subjects) and the prevalence was 86.4% for IgG and 2.1% for IgM [40]. An exclusive study conducted in western part of Ethiopia among pregnant women (201 subjects) reported 83.6% sero-prevalence of toxoplasmosis (81.1% for IgG, 2.5% for IgM) [41]. The major associated risk factors identified for infection of Toxoplasma Gondii in Ethiopia entail consumption of raw vegetable, presence of cat at home and contact with it, HIV infection status, pregnancy, history of eating raw meat (which is quite common in Ethiopia), and engagement in farming activities that involve contact of soil [40, 41]. Infection with HIV results in deterioration of immune system, exposing the body for various bacterial and viral infections. One of those infections is Toxoplasma Gondii, which is associated with HIV infection in Ethiopia and a study carried out in a hospital among infected (302 subjects) and non-infected (315 subjects) clients of HIV revealed that higher prevalence of toxoplasmosis among infected (93.3%) and male subjects than non-infected (86.3%) and female clients. According to sentinel survey (conducted 2009), the HIV prevalence among pregnant women was 3% [34], while the EDHS conducted in 2011 reported an HIV prevalence of 1.5% (1.9% for women and 1.0% for men) [14]. Therefore, despite unavailability of evidence that could show the congenital defects associated with HIV infection of pregnant women, HIV is prevalent among women in general and pregnant women specifically, who ultimately could be predisposed to various risk factors including Toxoplasmosis during pregnancy in which it is associated with congenital disorder of fetus.

### Prevalence of Birth Defects

Though information about rates of congenital disorders in Ethiopia are limited, available studies conducted in health facilities show that congenital heart diseases, cleft lip and palate, gastrointestinal malformations, club-foot, and others are common.

According to a study conducted in the teaching and referral hospital of Addis Ababa, out of 42, 986 live births, 64 were affected by cleft lip and/or palate, giving a rateof 1.5 per 1000 live births. Out of these cases, 4.8% had a family history of the same condition (1.6% of cleft lip, and 3.1% of cleft lip and palate), and almost 16% of all cases had other anomalies [42]. Another hospital based analysis demonstrated that among a total of 2, 281 surgical patients seen over five years, cleft-lip and palate accounted for 8% of all cases [43]. A similar study conducted in a hospital in the Northern part of Ethiopia revealed that out of 7489 infants admitted to pediatric wards over six years, 2.9% had congenital malformations including cardiac malformations, hernia, and gastrointestinal malformations. The same study reported that the mortality rate among infants with congenital malformations was 10.5% [44]. Another hospital based study reported that congenital abnormalities accounted for 2.1% of all obstructed labour cases [45]. Among 285 club-foot patients visiting hospital, 70.5% were diagnosed at birth and the majority of them (77%) came from the urban areas of Ethiopia [46]. The other most common congenital disorders that are estimated to be common in developing countries are hemoglobin disorders and G6PD deficiency, however, information pertaining to these disorders is unavailable in Ethiopia.

Management of congenital disorders is another hurdle for the developing countries in general and for Ethiopia in particular where health facilities are under equipped and specialized staff are few in number, and hence there are few health facilities that can manage congenital defects medically or surgically in Ethiopia. Recently, two hospitals began giving services related to congenital defects, and within two years of operation services a total of 1364 patients (63.1% male, 36.9% female) received these services, with 37.6% of the total were pediatric cases while 62.4% were reported to be aged of 14 or above. This study reported that hydrocephalus (35.5%), neural tube defects (27.5%) and neuro-trauma (9.7%) were the three major leading causes of admission to hospitals for surgical procedures among children. For adult patients, neuro-trauma (28.7%), chronic subdural hematomas (22.8%), spine and spinal cord diseases (21.2%) were the major neurosurgical causes for admission to hospitals. As some of the causes of neurosurgical cases are not life threatening, elective surgery is being performed in these hospitals. Many cases were still on waiting list as it would be impossible to manage all cases as they arrive at hospitals due to scarcity of resources and the fact that only these two hospitals are giving services to neurosurgical cases in Ethiopia. For instance, as reported by the same study, among pediatrics cases 81% of them had elective operations, and 19% of them went through emergency operations; while among adults, 55% and 45% of neurosurgical cases had emergency and elective operations, respectively [47]. Cases of congenital disorders might not seek medical care, particularly among the rural population who rarely visit health facilities and hence the magnitude of neurosurgical cases in the general population may be higher than that reported by this study from the two hospitals.

Most of the epidemiological studies on congenital disorders in Ethiopia were conducted in the few specialized hospitals in the country. Other population based studies were not directly related to the congenitaldisorders. Given that 90% of deliveries take place at home [14] in Ethiopia, studies conducted based on live births happening in health facilities could not give the true magnitude of the birth rates of congenital malformations. Hence, the rates reported from few health facilities could underestimate the burden of congenital diseases/disorders and only certain easily identifiable birth defects such as cleft lip and palate rates could be given. Genetic laboratory diagnosis is costly and unavailable for the general population. Though the health policy of Ethiopia is tilted towards prevention of diseases and promotion of health through decentralization of primary health care system to the grass root level of community [6], provision of prevention diagnostic and counseling services related to genetic diseases has not been well established.

## Conclusion

Some congenital disorders could be prevented at primary health care unit through screening and treatment before and during pregnancy, while others could be prevented by dietary supplementation during pregnancy. Existing limited information in Ethiopia shows that factors predisposing to congenital disorders are common: high folate deficiency and high iodine deficiency among women in the reproductive age; and high prevalence of syphilis and toxoplasmosis among pregnant women have been reported. Available epidemiological data on genetic diseases/disorders in Ethiopia is limited, and few health facility based studies conducted reveal that cleft lip and palate, congenital heart diseases, club-foot, and other malformations such as gastro-intestine malformations are common. There are no community based studies conducted directly related to genetic diseases/disorders that could accurately demonstrate the incidence and prevalence level of these conditions. Hence, to realize the organization of community genetic services program at the primary health care in Ethiopia, conducting community based epidemiological studies that could give a clear picture about predisposing factors and prevalence of congenital disorders is needed.

**Figure 1:**
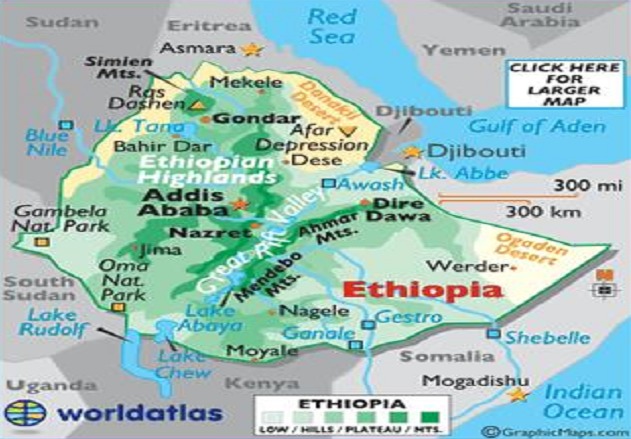
Map of Ethiopia (source World Atlas)
